# Status of Polymer Fused Deposition Modeling (FDM)-Based Three-Dimensional Printing (3DP) in the Pharmaceutical Industry

**DOI:** 10.3390/polym16030386

**Published:** 2024-01-30

**Authors:** Heba Iqbal, Queenie Fernandes, Sourour Idoudi, Renuka Basineni, Nashiru Billa

**Affiliations:** 1 Pharmaceutical Sciences Department, College of Pharmacy, QU Health, Qatar University, Doha P.O. Box 2713, Qatar; hi1309307@qu.edu.qa (H.I.); si1602796@student.qu.edu.qa (S.I.); renuka.basineni@qu.edu.qa (R.B.); 2 College of Medicine, QU Health, Qatar University, Doha P.O. Box 2713, Qatar

**Keywords:** amorphous solid dispersion, fused deposition modeling, solubility, polymer, pharmaceutical, three-dimensional printing

## Abstract

Additive manufacturing (AM) or 3D printing (3DP) is arguably a versatile and more efficient way for the production of solid dosage forms such as tablets. Of the various 3DP technologies currently available, fused deposition modeling (FDM) includes unique characteristics that offer a range of options in the production of various types of tablets. For example, amorphous solid dispersions (ASDs), enteric-coated tablets or poly pills can be produced using an appropriate drug/polymer combination during FDM 3DP. The technology offers the possibility of evolving personalized medicines into cost-effective production schemes at pharmacies and hospital dispensaries. In this review, we highlight key FDM features that may be exploited for the production of tablets and improvement of therapy, with emphasis on gastrointestinal delivery. We also highlight current constraints that must be surmounted to visualize the deployment of this technology in the pharmaceutical and healthcare industries.

## 1. Introduction

Three-dimensional printing (3DP) is an additive manufacturing (AM) technique whereby material is gradually added up, layer by layer, to construct a 3D geometric representation of a digitized imagery [1]. Since its inception in the 1980s, AM technology has evolved exponentially due to its unique features, such as an amenability for constructing complicated geometries with composite materials mimicking body parts, organs or pharmaceutical dosage forms, therefore eliminating traditional manufacturing processes that are time-consuming and convolute [2]. Thus, 3DP has an untapped potential in biomedical, pharmaceutical and industrial applications [2]. The 3DP market has boomed over the last decade, largely buoyed by cost-effectiveness, increased printing speed and precision on printed prototypes [3]. Therefore, for industrial production processes aimed at increasing industrial efficiency, 3DP techniques offer a formidable scope for product design and expansion. Three-dimensional printing technology is utilized in the automotive, aerospace, medical, food, electrical and construction industries [4]. Its application in the pharmaceutical industry has only begun to gain traction within the last decade due to the attributes presented above and, especially, the possibility for customization and affordability [5,6,7,8,9]. Various dosage forms, such as orally administered tablets [10], transdermal patches and microneedles have been successfully fabricated using AMTs [11]. Furthermore, AMTs have also been implicated for clinical use, including cardiology [12], neurosurgery [13], otolaryngology [14], pulmonology [15], podiatry [16], gastroenterology [17] and radiotherapy [18]. In light of its potential applications within the pharmaceutical industry, 3DP technology is likely to advance the sector’s scope of products output and applications, especially in the realm of personalized medicines [19].

Pharmaceutical production methods like capsule filling and tableting have advanced in the last few decades, especially in the provision of modified drug-release profiles through novel drug-delivery formulations, including polymeric matrices, nanoparticles, functionalized liposomes and biomimetic particles [20]. However, these formulations go as far as to provide doses to patients based on label claims. They do not account for the variations in dosage requirements amongst patients imposed by genetic or metabolic predispositions and, therefore, cannot be tuned for the requirements of personalized medicines. This is mainly due to process restrictions within conventional production modalities. Absorption of active pharmaceutical ingredients (APIs) following oral administration of conventional dosage forms is associated with a variability in the release of APIs from the dosage form and unpredictable pharmacokinetics. AMT provides scope for the design of patient-centered dosage forms with programmable release capable of minimizing unpredictability in the absorption and maximizing therapy [20].

The first and only 3D-printed tablet currently on the market (Spritam^®^ by Aprecia Pharmaceuticals, Blue Ash, OH, USA) received Food and Drug Administration (FDA) approval in 2015. The tablet is administered via the oral route and is used in patients with dysphagia [21]. Liquid dosage forms may also be used in dysphagic patients; however, these offer diluted dosing and promote instability of the API [22,23]. Interestingly, T19 is another 3D-printed drug produced by Triastek, a Chinese pharmaceuticals and 3D printing technology firm, that has received an investigational new drug (IND) approval from the FDA. T19 is been designed for treatment in rheumatoid arthritis [24].

**Figure 1:**
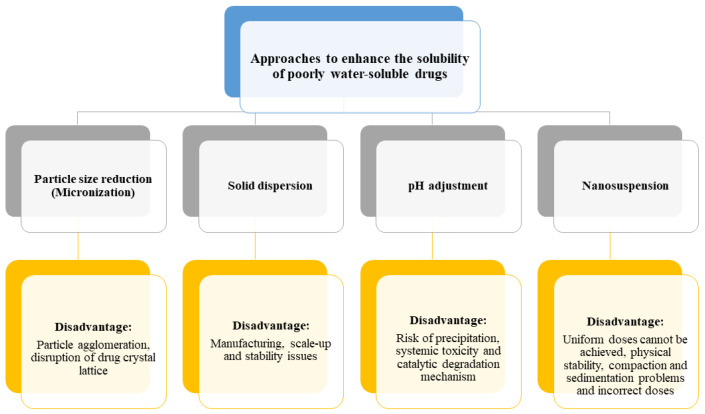
Solubility-enhancing approaches and disadvantages.

Several approaches have currently been applied to improve the solubility of drugs, including particle size reduction, nanosuspension, salt formation, pH adjustment, use of surfactants and use of amorphous solid dispersions (ASDs) [25,26,27,28,29,30]; however, solubility enhancement techniques tag along peculiar constraints [31,32], as presented in Figure 1.

Three-dimensional printing technology has emerged in recent years as a possible tool for the production of ASDs, with the inclusion of variable doses of APIs in solid dosage forms [33]. In the context of customizing 3DP technology for individual patient needs, this dual approach of 3DP and ASD may be useful at dispensaries and pharmacies, with scope for the improvement in solubility of APIs and the production of variable doses of medications [33]. Furthermore, the approval of Spritam^®^ has set the precedence for the utilization of 3DP in the manufacture of newer drug-delivery systems [34].

Three-dimensional printing offers different types of feed mechanisms (technologies) such as material jetting, powder bed fusion, direct energy deposition, binder jetting, light photopolymerization and extrusion. These types of 3DP technologies enable the 3D printing of materials such as fluids, waxes, powder and solids [35]. Thus, there is flexibility in the choice of the appropriate material as per requirement.

Aptly, AM techniques offer robust and potent platforms that can be largely employed by pharmaceutical companies for the manufacture of various doses and dosage forms as alluded to above. However, fused deposition modeling (FDM) is an extensively used extrusion-based approach, with consequential outcomes in the pharmaceutical industry due to the similarity to other extrusion techniques already utilized in the industry, i.e., hot melt extrusion (HME) [36]. Moreover, FDM offers the possibility of utilizing biodegradable thermoplastic polymers commonly employed in the formulation of drug products [37,38]. FDM technology is affordable, amenable to modifications, and is simple and may be adapted for desktop usage [39]. Therefore, there is potential for its adoption and evolution in pharmacies and dispensaries toward the provision of personalized medicines [40,41]. In this review, we expound the potential of the FDM 3D printing technique in the pharmaceutical industry along with the challenges it faces.

## 2. Key Elements of Fused Deposition Modeling (FDM)

In FDM, the print material, usually a thermoplastic filament, is deposited selectively onto a build platform as it melts and oozes out of a nozzle or orifice. The platform and nozzle move in synchrony to allow the layer-by-layer construction of a 3D model [42]. Materials including plastic prototypes and low-volume functionality components can be fabricated using FDM extrusion-based approaches. For instance, FDM is the most extensively used extrusion-based approach for modeling, prototyping and fabrication [43,44]. FDM 3D printing utilizes a variety of thermoplastic polymers including polylactic acid (PLA), acetonitrile butadiene styrene (ABS), polypropylene (PP) and polyvinyl alcohol (PVA). These polymers facilitate fabrication processes and the fabricated object may provide feedback on the efficiency of the filament for the printing [45].

The production of a filament for the FDM process can be achieved through HME, whereby the polymer is heated and squeezed through an extruder to produce the filament. Once the filament is formed, a 3D-printed object may be fabricated using an FDM 3D printer [36,46].

With regard to the potential applications of FDM by the pharmaceutical industry and pharmacies, the need for incorporation of an API in the filament is very crucial. An API may be incorporated in the filament via impregnation (IMP) after the filament is formed or during HME of the filament [47]. However, API loading via IMP after formation of the filament is inefficient because it results in very low drug loads. Maximum drug loading is achievable through incorporation during HME [47,48]. This approach also allows the incorporation of other additives, including plasticizers or polymers (as in the formation of ASDs) [48]. As previously reported, HME may also be used to produce ASDs of poorly water-soluble drugs, whereby the API is presented in an amorphous configuration [48]. This amorphous configuration of the API ensures improved solubility.

**Figure 2:**
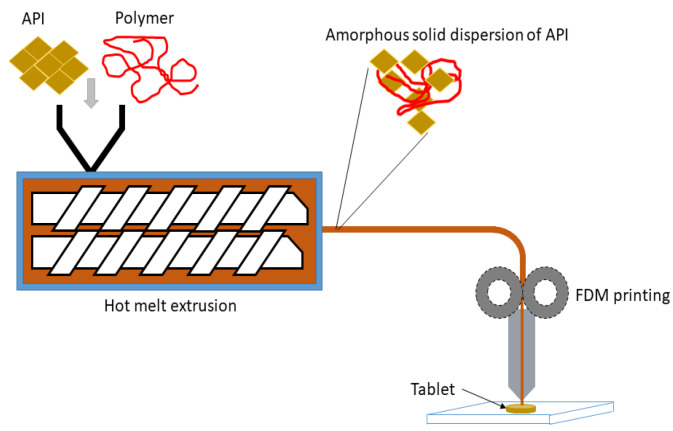
Polymer fused deposition modeling (FDM)-linked hot melt extrusion (HME).

The HME mixer also ensures that the API is homogenous within the extruded filament. Mixing can be carried out on a double or single screw mixer [46]. Subsequently, the fabricated filament is fed into the FDM printing machine to print a tablet [48], as shown in Figure 2.

The coupling of HME with FDM-based 3DP allows for the production of pharmaceutical-grade filaments for the printing of medicines [49]. In this light, HME is favored for obtaining drug-loaded filaments, which are used as starting materials for the 3D printing of tablets and other dosage forms [50]. The use of HME to produce filaments with defined shapes and properties is crucial for the success of 3D printing in pharmaceutical applications [51]. The continuous and cost-efficient nature of HME makes it an attractive manufacturing process for drug-delivery systems [52]. Moreover, the use of HME in combination with 3D printing technology also supports the development of personalized medicine and targeted drug-delivery systems [53]. The production of filaments through HME is a critical step in the fabrication of 3D-printed dosage forms, enabling the incorporation of drugs into the matrix and ensuring the smooth structure of the filaments for successful 3DP [53].

However, the potential applications of HME coupled with FDM-based 3DP may be limited, especially in the bioprinting of thermolabile drugs. However, alternative strategies have been suggested in order to overcome such limitations. One approach involves reducing the FDM printing temperature to accommodate low-melting and thermolabile drugs, as demonstrated by a certain study [54]. Furthermore, the use of natural products in the preparation of 3D-printed drug-delivery systems has been investigated, providing a valuable potential for the fabrication of thermolabile drug-containing tablets via FDM [55]. In addition, inkjet printing of thermolabile model drugs onto FDM-printed substrates has also been explored, offering a potential alternative strategy for drug formulation and evaluation [56]. Moreover, the use of polymer blends to improve the printability and to regulate drug release from pharmaceutical solid dispersions prepared via FDM 3D printing has been investigated, indicating a promising approach to address the challenges associated with printing thermolabile drugs [57]. These alternative strategies demonstrate the ongoing efforts to overcome the limitations of FDM-based printing of thermolabile drug substances, offering potential solutions for the fabrication of patient-tailored dosage forms and drug-delivery systems.

Similarly, other 3D printing techniques such as selective laser sintering (SLS) and stereolithography (SLA) have also been evaluated as suitable alternatives to FDM-based printing. These techniques involve the loose packaging of polymers, ultimately giving rise to porous structures that serve as enhanced drug-delivery systems. However, due to the use of high-energy lasers in such manufacturing processes, damage to especially sensitive drugs is a huge possibility [58]. Therefore, these techniques may not be frequently employed in the manufacturing of drug-loaded formulations. Moreover, in comparison to FDM-based 3DP, they may prove to be less cost-efficient. For example, FDM processes require lower initial investment and operational costs, as compared to SLA. FDM printers are generally more affordable, and the materials used in FDM are often cheaper than SLA resins. In addition, the binder-jetting 3D printing technique can be used to produce amorphous dosage forms for heat-sensitive drugs with high speed using a liquid binding agent to bond thin layers of solid powder [58].

## 3. Polymers Utilized in FDM-Based 3DP

Commonly used filaments in FDM 3DP techniques include acrylonitrile butadiene styrene (ABS) and polylactic acid (PLA) [4]. ABS is a thermoplastic polymer manufactured from petroleum, through combining acrylonitrile, butadiene and styrene. Owing to its toughness and impact resistance, it has found numerous applications in the automotive industry and in the production of marine components and toys [5]. However, due to its nonbiodegradability and mild toxicity, it is not the preferred choice in pharmaceutical industries [3].

In comparison, polylactic acid (PLA) is another thermoplastic polymer that has an advantage over ABS, due to it being biodegradable [5]. Accordingly, it has received approvals from both the FDA and the European regulatory authorities for its application in the medical and food industries [59]. The utilization of PLA is linked to several advantages, including biocompatibility [60].

Likewise, owing to the growing interest in FDM-based 3DP, many studies have investigated the use of various other polymers in the fabrication of filaments for use in FDM-based 3D printing. Consequently, a particular study demonstrated the potential of pharmaceutical-grade polymers such as polyvinylpyrrolidone (PVP), polyvinyl alcohol (PVA), Kollicoat^®^ IR (KIR), Soluplus^®^ (SLP), polyethylene oxide (PEO), hydroxypropyl methyl cellulose (HPMC), Eudragit^®^ L (ERD L), Eudragit^®^ RL (ERD RL) and ethyl cellulose (EC) as filaments for 3DP [61]. Interestingly, this study reported that drug release is largely dependent on the type of polymers used in the manufacture of the filament and differs based on their water solubility. For example, polymers such as PEO and KIR offer an almost immediate drug release, while on the other hand, HPMC, PVA and SLP are used for sustained or extended drug release [61]. Conversely, the use of poorly permeable hydrophobic polymers such as EDR RL and EC often results in an extremely slow release of the drug [61]. Similarly, other agents, like “Ticagrelor”, a blood-thinning drug, and “Tacrolimus”, a macrolide inhibitor of calcineurin used in organ transplantations, have also been developed using 3DP techniques [62,63]. In addition, another slow-release drug, in the form of pH-responsive tablets for colon drug-delivery applications, have been developed through the use of 3DP technology [64]. Evidently, an array of polymers is currently available for use in the application of FDM-based 3DP in pharmaceutical industries. However, the choice of polymer is tightly governed by the specification of the type of drug, its biological target and the duration of the therapy [61].

## 4. FDM 3D Printing and Amorphous Solid Dispersions (ASDs)

**Figure 3:**
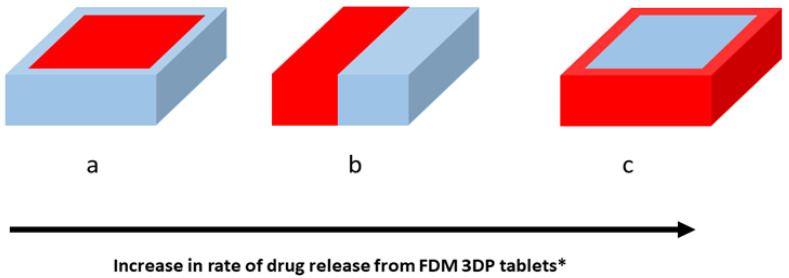
Red shade: drug in soluble polymer (PVA); blue shade: water-insoluble PLA filler only. (**a**) ASD surrounded by insoluble PLA in dosage form; (**b**) ASD adjacent to insoluble PLA in dosage form; (**c**) Insoluble PLA surrounded by ASD. * (Reproduced with permission from reference [59]).

ASDs may be defined as a solid dispersion that involves the melting of a solid mixture of API and a suitable vehicle, usually polymers that form eutectic mixtures [51,65]. The polymer or “solvent”, interferes with the ordered arrangement of the crystalline API and thereby transforms the mixture into an amorphous solution [51,52,65,66]. The use of FDM in conjunction with ASDs of APIs with low solubility has shown that 3D-printed tablets derived as such show considerable improvement in the solubility of the API [67]. However, the type of polymer used in the printing is very crucial. In a particular study [57] on the formation of felodipine ASDs using FDM 3DP, they observed that the drug-release rate can be altered by varying its miscibility in the polymer blend. The polymer blending technique is an efficient formulation strategy widely used in the plastic and polymer industries to improve the processibility of the material [57]. In addition to the miscibility of the API in the polymer blend, the printability of the blend using FDM is equally crucial. A separate mixture of Eudragit EPO and Soluplus with PEG (polyethylene glycol), PEO (polyethylene oxide) and/or Tween 80 resulted in excellent printability of the blends, as opposed to only Eudragit EPO or Soluplus used alone, which exhibit poor fluidity as well as a high-melt viscosity. This is despite the fact that Eudragit EPO and Soluplus are extensively used in the HME, but are clearly not suitable for FDM 3DP [57]. Furthermore, the ratio of the API to the excipient in the filament can also affect the printability of the filament using FDM. In a study by Kissi et al., HME-extruded filaments containing naproxen were discovered to be amorphous and by increasing the API ratio from 0–10% to 10–20%, the filament brittleness was reduced and printability improved without a compromise on ASD stability. This was due to the plasticizing effect imparted to the filament by the API [68]. In another study, Tan et al. also showed that the configuration of the API in the filament and FDM-printed tablets remained amorphous and the polymeric solvent controls the ductility and flexibility of the formed filament, which, in turn, affects the efficiency of printing using FDM [69]. In the same study, the dissolution behavior of theophylline from 3DP tablets using FDM using hydroxyl propyl cellulose (HPC), polyethylene glycol (PEG) and Eudragit^®^ RL/PO showed that both HPC and PEG tablets were fully disintegrated/dissolved, but not the Eudragit^®^ RL/PO tablets, retaining its shape because of the insoluble nature of the Eudragit matrix [69]. Consequently, the rates of theophylline release from the HPC and PEG tablets were higher than from the Eudragit^®^ RL/PO [69]. The retention of tablet shape or disintegration of matrices during API release will also dictate the release mechanism. In the case of the Eudragit^®^ RL PO, the matrices are permeable but not soluble; thus, release is likely a diffusion-mediated profile. Furthermore, biodegradability of a polymer in physiologic media will also impact the rate of the release mechanism of the API from the printed tablets; for example, polylactic acid (PLA) has a degradation half-life of approximately 210 days, hence the biodegradation-mediated release of API will be a slow process [70,71]. Tagami et al. showed that by increasing the hydrophobic filler component consisting of PLA and decreasing the hydrophilic component containing a hydrophilic API, e.g., calcein, a slow release profile was obtained and vice versa from the FDM polymer composite [72], as illustrated in Figure 3a–c (reproduced with permission). Thus, through careful choice of polymer blends, and printing geometries, we can modulate the rates of API release from FDM-printed ASD. In a related study, Jamróz et al., 2018 [73] separately utilized Kollicoat^®^ IR (water-soluble polymer) or PLA (water insoluble) to construct tablets using FDM, whereby the tablets formulated with the PLA polymer presented a prolonged release profile of up to 70% after 6 h. On the other hand, the tablet constructed with Kollicoat^®^ IR showed 90% of drug release within 45 min [73]. This shows that polymer combinations can be very useful for tailoring the drug release from the FDM 3D-printed tablets [73,74].

However, some single water-soluble polymers are not printable; for example, Kollidon^®^ VA64 (PVP-VA) due to brittleness [75]. Furthermore, some hydrophobic polymers such as PLA impede API release from the FDM 3D-printed tablets, which is exacerbated when the API is completely miscible in the polymer [76].

In addition, alternate strategies to FDM-based 3DP have been developed in order to eliminate the need to produce filaments for subsequent fabrication into 3DP tablets using FDM; for example, direct powder extrusion (DPE) may be used to prepare ASDs [77], whereby ASD/3D-printed tablets are produced in a single step, with effective improvement in the solubility of a poorly soluble API [77]. However, when compared to FDM, DPE is less reliable in the formation of ASDs due to the possibilities of recrystallization of API from the ASD. On the other hand, DPE is useful in the formation of ASDs of thermolabile APIs [39].

## 5. FDM-Printed Tablets for Targeted Gastrointestinal Drug Delivery

Since FDM-printed tablets are almost certainly destined for oral administration, this section is dedicated to relevant pharmaceutical technologies that may be employed in conjunction with FDM designs for effective gastrointestinal drug deployment. In addition to the possibilities of forming ASDs, the FDM technique may be used to produce individualized doses to match the severity of a disease or patient predispositions [78]. Combined ASD/FDM technologies can potentially be used for the production of poly pills in chronically ill patients, with the added provision of improving the solubility of APIs [78]. With regards to the oral administration of 3DP tablets, it is important to recognize the physiological and anatomical constraints along the gastrointestinal tract as well as features that can be exploited to maximize absorption. The enzymatic milieu, acidic environment of the stomach and neutral to alkaline pH of the small intestine and colon all provide challenges and opportunities for successful deployment of APIs from FDM-printed tablets [79]. In this regard, the careful selection of polymer/excipients in FDM design and production can be tuned to achieve targeted drug delivery.

For example, despite the low absorptive surface area of the colon, it may be optimal for the absorption of many API drugs due to low enzyme/proteolytic activity and extended transit of dosage forms [80]. Thus, colon-targeted delivery of APIs is recognized as a potential strategy for systemic deployment of protein and gene products [80]. FDM design may incorporate enteric polymers such as Eudragit^®^ FS30 D or cellulose acetate phthalate as a printed coating, which dissolves at a pH of about 7, and is therefore ideal for shielding acid-labile drugs from the acidic pH of the stomach or for a colon-targeted delivery of therapeutics. The use of these polymers as coatings in conventional tablet dosage forms is well established. However, FDM printing technologies provide scope for the incorporation of additional polymers for pharmaceutical or therapeutic applications in a relatively simpler manner. Finally, there is more flexibility for the evolution of FDM tablet designs aimed at achieving targeted gastrointestinal delivery [81,82].

## 6. FDM-Based Bioprinting of Implant Transplantation Devices and Prosthetics

The applications of AMT in the biomedical sector are diverse. In this light, the role of FDM-based 3DP in personalized medicine is fundamental because customized patient therapy is growing into the future of targeted medicine.

The bioprinting of tissues and organs for suitable transplantation into the host is one such application with a high demand. This process utilizes a digitally guided pipette in order to layer living cells to artificially engineer living tissue according to a prespecified blueprint [83]. Currently, bioprinting has been used to develop tissues of the bone such as cartilaginous structures, and heart tissues such as vascular grafts, in addition to multilayered skin grafts and tracheal splints [84]. Interestingly, some studies [34,85] have also explored the embedding of drug-delivery systems inside the generated implants in order to facilitate enhanced surgical recovery and complete restoration of intended activity. In recent times, implant dosage forms engineered through the utilization of 3DP platforms is indeed gaining momentum for its crucial application in the pharmaceutical industry. Interestingly, this technology is able to produce implants in both micro- and macro-architecture settings. Accordingly, a particular study demonstrated that drug implants fabricated through 3DP have more clear advantages, as compared to implants engineered through conventional compressing-based methodologies [86]. This is particularly because 3DP techniques facilitate the generation of implants with a higher porosity, thereby offering a complex and sophisticated release profile that is difficult to achieve through other conventional methods. In this light, a variety of implants have been generated using 3DP technologies, namely, implants with the pulsed, bimodal, immediate, sustained, delayed and complex drug-release profiles [86,87].

Similarly, artificially generated organoids and tissues generated through the use of 3DP platforms have also found their application in medical research, due to their ability to mimic organs in vitro, thus offering an economical and sustainable experimental platform [83]. The use of medical models is fundamental in medical training, pre- and postoperative planning as well as in patient education [84,88]. However, more recently, through the use of AMTs, it is now also possible to engineer life-sized organ implants and anatomical models that have found their application in surgical practice and training, as well as in assisting medical diagnosis [89,90,91,92,93]. These models are often created from two-dimensional images like X-rays, CT scans or MRIs that are generally patient-specific, thereby offering ideal and realistic structures of intricate anatomical parts of the human body. In addition, 3DP has also found its application in the generation of tissue scaffolding and mechanical bone replicas [94,95]. Distinct techniques like electro-spinning, freeze-drying, emulsification and solvent casting are often employed in the development of tissue scaffolds [83]. Biomanufacturing is yet another technology that utilizes AMTs in combination with tissue engineering [96]. This process is often used to generate biocompatible structures to compensate for osteo defects [97].

FDM-based 3DP has also found its application in the development of medical devices that significantly enhance surgical and clinical procedures. It is now utilized to construct orthopedic instruments, and dental and surgical guides that can follow the patient’s unique anatomy with high levels of precision [98]. Such tools are purposed for application in complex and intricate surgeries, thus providing enhanced safety to the patient [99]. These are often engineered according to patient-customized dimensions, such as in drilling guides [100]. Also, in the area of dental practice, FDM-based 3DP has been exploited in the development of dental pieces like bridges and crowns [101,102]. Currently, the generation of customized instruments for use in dental surgery are commonly preferred [103,104].

Another stratum of biomedicine where the use of AMTs is imperative is in the development of prosthetics. Prosthetic limbs can be engineered and personalized to the fit of the patient. Three-dimensional printing is extremely efficient in the generation of customized prostheses like cranial, maxillofacial and mandible implants, thus helping in the resolution of various orthopedic impediments [105]. For example, a particular study [106] used 3DP to design and develop joint prostheses that have been engineered on the basis of surgically resected tibial osteosarcomas. Similarly, another study [107] discussed the implantation of femur modular prosthesis in a patient with osteosarcoma that was generated through the use of AMT. The study stated that the patient was able to attain complete painless recovery with perfect weight-bearing capacity. The process of development of prosthetics usually begins with medical imaging and segmentation, followed by three-dimensional scanning and finally 3D modeling [108]. In certain cases, manufacturing techniques involving AMTs can also be computer numerical control technologies [109].

## 7. Challenges

There are some significant challenges associated with the use of FDM. This technique may be best suited for small-scale prototyping, although larger printers are in use in various industries. In this light, although FDM-based 3D printing may identify as an easily operable technique with less postprocessing requirements, it is also commonly associated with certain limitations [110]. First, it is a relatively slow and expensive method of production of final articles owing to the limited availability and high cost of raw materials [111]. Second, it often requires skilled labor in order to achieve precise and accurate end-products. Consequently, despite the fact that materials used in 3DP may last longer as compared to the conventional pharmaceutical and industrial manufacturing procedures, a higher level of precision is often demanded in order to achieve desirable results, thereby slowing down the duration of the process [112]. However, in foresight, a gradual decline in the costs of the materials and machinery is expected in the near future.

Moreover, in comparison to traditional processes of drug manufacture, FDM-based 3DP is led by a three-dimensional modeling of drugs that is largely software based [113]. As a result, an infinite amount of product variability is achievable without an additional cost, as compared to conventional technologies of drug manufacture that demand a detailed architecture of products and multistep processes for customized end products [113]. Thus, FDM-based 3DP may be the best-suited option for personalized therapies, where drugs are often produced/dispensed in relatively smaller quantities.

Further, under certain circumstances, such product schemes identify with a tradeoff for lack of precision. There is also inaccuracy in the nozzle temperature, in effective solidification and in poor layer adhesion [114]. FDM produces low-resolution finishing, and twisting/wrapping problems [115]. Thus, there is the need for post-manufacture processing, or worse, structural demolition to achieve the desired configuration of the prototype [114]. Moreover, the volume capacity of the printer sets a restriction on the size of the printed object [114].

With regards to FDM-based 3DP for pharmaceutical dosage forms, thermolabile drugs are likely to be degraded during printing [116]. Inadequate API loading in filaments and, subsequently, in printed tablets is a key constraint in the use of FDM leading slow rates of API release [116]. On the other hand, an increased API content modifies the crystallographic, thermal and rheological properties of the filament, with the possibility of rendering them unprintable [116]. Since FDM is a mechanical process, failure within moving parts can affect the printing process [117]. Crucially, there is only a handful of suitable thermoplastic polymers suited for use in FDM 3DP pharmaceutical dosage forms [72]. Use of a hydrophobic polymer such as PLA may cause a slow drug release from printed tablets. Innovative designs aimed at increasing the surface area of printed tablets exposing the drug to the media only marginally improved the rate of release [118]. These constraints have negatively impacted the utilization of FDM in the pharmaceutical industry [118]. However, with key advances in polymer chemistry and pharmaceutical technology, it is the view of the authors that these constraints are not insurmountable.

## 8. Conclusions

We conclude that FDM-based 3DP offers a huge potential to the pharmaceutical industry for the fabrication of a variety of solid dosage forms, particularly those intended for gastrointestinal delivery. It is versatile and permits the incorporation of several pharmaceutical excipients in relatively fewer production steps. The formation of ASDs is noteworthy, where improvement in the solubility of APIs is an added output. Furthermore, the technique provides a framework for polymer combinations in tablet designs, including gastrointestinal targeted. The FDM 3D printing technique also provides insights into the production of personalized medicines due to the efficiency in production. Notwithstanding, there are regulatory and production constraints, but these are not insurmountable. It is our view that research should focus on the production of biocompatible/biodegradable thermoplastic polymers that also promote API release from printed tablets, because this may shorten the trajectory to the realization of FDM 3DP tablets on the market.

## Data Availability

Data are contained within the article.

## References

[1] Mwema F.M., Akinlabi E.T. (2020). Basics of Fused Deposition Modelling (FDM). Fused Deposition Modeling.

[2] Gibson I., Rosen D., Stucker B. (2015). Additive Manufacturing Technologies.

[3] Yan Q., Dong H., Su J., Han J., Song B., Wei Q., Shi Y. (2018). A Review of 3D Printing Technology for Medical Applications. Engineering.

[4] Shahrubudin N., Lee T.C., Ramlan R. (2019). An Overview on 3D Printing Technology: Technological, Materials, and Applications. Procedia Manuf..

[5] Banks J. (2013). Adding Value in Additive Manufacturing: Researchers in the United Kingdom and Europe Look to 3D Printing for Customization. IEEE Pulse.

[6] Mertz L. (2013). Dream It, Design It, Print It in 3-D: What Can 3-D Printing Do for You?. IEEE Pulse.

[7] Kim K., Ratri M.C., Choe G., Nam M., Cho D., Shin K. (2020). Three-Dimensional, Printed Water-Filtration System for Economical, on-Site Arsenic Removal. PLoS ONE.

[8] Rankin T.M., Giovinco N.A., Cucher D.J., Watts G., Hurwitz B., Armstrong D.G. (2014). Dimensional Printing Surgical Instruments: Are We There Yet?. J. Surg. Res..

[9] Ursan I.D., Chiu L., Pierce A. (2013). Three-Dimensional Drug Printing: A Structured Review. J. Am. Pharm. Assoc..

[10] Sun Y., Soh S. (2015). Printing Tablets with Fully Customizable Release Profiles for Personalized Medicine. Adv. Mater..

[11] Ye Y., Yu J., Wang C., Nguyen N., Walker G.M., Buse J.B., Gu Z. (2016). Microneedles Integrated with Pancreatic Cells and Synthetic Glucose-Signal Amplifiers for Smart Insulin Delivery. Adv. Mater..

[12] Vukicevic M., Mosadegh B., Min J.K., Little S.H. (2017). Cardiac 3D Printing and its Future Directions. JACC Cardiovasc. Imaging.

[13] Thawani J., Randazzo M., Pisapia J., Singh N. (2016). 3D printing in neurosurgery: A systematic review. Surg. Neurol. Int..

[14] Crafts T.D., Ellsperman S.E., Wannemuehler T.J., Bellicchi T.D., Shipchandler T.Z., Mantravadi A.V. (2016). Three-Dimensional Printing and Its Applications in Otorhinolaryngology–Head and Neck Surgery. Otolaryngol. Neck Surg..

[15] Guibert N., Mhanna L., Didier A., Moreno B., Leyx P., Plat G., Mazieres J., Hermant C. (2018). Integration of 3D Printing and Additive Manufacturing in the Interventional Pulmonologist’s Toolbox. Respir. Med..

[16] Williams C., James A., Chae M.P., Hunter-Smith D.J. (2015). 3D Printing in Clinical Podiatry: A Pilot Study and Review. J. Foot Ankle Res..

[17] Jeon H., Kang K., Park S., Kim W.D., Paik S.S., Lee S.-H., Jeong J., Choi D. (2017). Generation of Multilayered 3D Structures of HepG2 Cells Using a Bio-printing Technique. Gut Liver.

[18] Su S., Moran K., Robar J.L. (2014). Design and Production of 3D Printed Bolus for Electron Radiation Therapy. J. Appl. Clin. Med. Phys..

[19] Chen G., Xu Y., Kwok P.C.L., Kang L. (2020). Pharmaceutical Applications of 3D Printing. Addit. Manuf..

[20] Mohammed A., Elshaer A., Sareh P., Elsayed M., Hassanin H. (2020). Additive Manufacturing Technologies for Drug Delivery Applications. Int. J. Pharm..

[21] Reddy C.V., Balamuralidhara V., Venkatesh M., Kumar T.P. (2020). First FDA Approved 3D Printed Drug Paved New Path for Increased Precision in Patient Care. Appl. Clin. Res. Clin. Trials Regul. Aff..

[22] Wright D., Begent D., Crawford H. (2017). Medication Management of Adults with Swallowing Difficulties. Consensus Guideline on the Gedication Ganagement of Adults with Swallowing Difficulties.

[23] Pamudji J.S., Mauludin R., Nurhabibah (2014). Influence of β-Cyclodextrin on Cefixime Stability in Liquid Suspension Dosage Form. Procedia Chem..

[24] Everett H. Triastek Receives FDA IND Clearance for 3D Printed Drug to Treat Rheumatoid Arthritis. https://3dprintingindustry.com/news/triastek-receives-fda-ind-clearance-for-3d-printed-drug-to-treat-rheumatoid-arthritis-184159/.

[25] Sigfridsson K., Lundqvist A.J., Strimfors M. (2009). Particle Size Reduction for Improvement of Oral Absorption of the Poorly Soluble Drug UG558 in Rats during Early Development. Drug Dev. Ind. Pharm..

[26] Doke V.V., Khutle N.M., Sharma M., Gupta K. (2022). Solubility Enhancement of Poorly Soluble Drug Ezetimibe by Developing Self Nano Emulsifying Drug Delivery System. Indian J. Sci. Technol..

[27] Putra O.D., Umeda D., Fujita E., Haraguchi T., Uchida T., Yonemochi E., Uekusa H. (2018). Solubility Improvement of Benexate through Salt Formation Using Artificial Sweetener. Pharmaceutics.

[28] Choudhary A.N., Nayal S. (2019). A Review: Hydrotropy a Solubility Enhancing Technique. Pharma Innov. J..

[29] Hart M.L. (2013). Brief Overview of Various Approaches to Enhance Drug Solubility. J. Dev. Drugs.

[30] Dalvi P.B., Gerange A.B., Ingale P.R. (2015). Solid dispersion: Strategy to enhance solubility. J. Drug Deliv. Ther..

[31] Vemula V.R., Lagishetty V., Lingala S. (2010). ChemInform Abstract: Solubility Enhancement Techniques. Int. J. Pharm. Sci. Rev. Res..

[32] Pawar S.S., Dahifale B.R., Nagargoje S.P., Shendge R.S. (2017). Nanosuspension Technologies for Delivery of Drugs. Nanosci. Nanotech. Res..

[33] Trenfield S.J., Januskaite P., Goyanes A., Wilsdon D., Rowland M., Gaisford S., Basit A.W. (2022). Prediction of Solid-State Form of SLS 3D Printed Medicines Using NIR and Raman Spectroscopy. Pharmaceutics.

[34] Prasad L.K., Smyth H. (2015). 3D Printing Technologies for Drug Delivery: A Review. Drug Dev. Ind. Pharm..

[35] Kamran M., Saxena A. (2016). A Comprehensive Study on 3D Printing Technology. MIT Int. J. Mech. Eng..

[36] Patil H., Tiwari R.V., Repka M.A. (2016). Hot-Melt Extrusion: From Theory to Application in Pharmaceutical Formulation. AAPS PharmSciTech.

[37] Zema L., Melocchi A., Maroni A., Gazzaniga A. (2017). Three-Dimensional Printing of Medicinal Products and the Challenge of Personalized Therapy. J. Pharm. Sci..

[38] Thakkar R., Thakkar R., Pillai A., Ashour E.A., Repka M.A. (2020). Systematic Screening of Pharmaceutical Polymers for Hot Melt Extrusion Processing: A Comprehensive Review. Int. J. Pharm..

[39] Melocchi A., Briatico-Vangosa F., Uboldi M., Parietti F., Turchi M., von Zeppelin D., Maroni A., Zema L., Gazzaniga A., Zidan A. (2020). Quality Considerations on the Pharmaceutical Applications of Fused Deposition Modeling 3D Printing. Int. J. Pharm..

[40] Skowyra J., Pietrzak K., Alhnan M.A. (2015). Fabrication of Extended-Release Patient-Tailored Prednisolone Tablets via Fused Deposition Modelling (FDM) 3D Printing. Eur. J. Pharm. Sci..

[41] Pietrzak K., Isreb A., Alhnan M.A. (2015). A Flexible-Dose Dispenser for Immediate and Extended Release 3D Printed Tablets. Eur. J. Pharm. Biopharm..

[42] Friel R.J. (2015). Power Ultrasonics for Additive Manufacturing and Consolidating of Materials. Power Ultrasonics.

[43] Praveena B.A., Lokesh N., Buradi A., Santhosh N., Praveena B.L., Vignesh R. (2021). Comprehensive Review of Emerging Additive Manufacturing (3D Printing Technology): Methods, Materials, Applications, Challenges, Trends and Future Potential. Mater. Today Proc..

[44] Zarek M., Layani M., Cooperstein I., Sachyani E., Cohn D., Magdassi S. (2015). 3D Printing of Shape Memory Polymers for Flexible Electronic Devices. Adv. Mater..

[45] Kristiawan R.B., Imaduddin F., Ariawan D., Ubaidillah, Arifin Z. (2021). A Review on the Fused Deposition Modeling (FDM) 3D Printing: Filament Processing, Materials, and Printing Parameters. Open Eng..

[46] Maniruzzaman M., Boateng J.S., Snowden M.J., Douroumis D. (2012). A Review of Hot-Melt Extrusion: Process Technology to Pharmaceutical Products. ISRN Pharm..

[47] Thanawuth K., Sutthapitaksakul L., Konthong S., Suttiruengwong S., Huanbutta K., Dass C.R., Sriamornsak P. (2021). Impact of Drug Loading Method on Drug Release from 3D-Printed Tablets Made from Filaments Fabricated by Hot-Melt Extrusion and Impregnation Processes. Pharmaceutics.

[48] Long J., Gholizadeh H., Lu J., Bunt C., Seyfoddin A. (2017). Application of Fused Deposition Modelling (FDM) Method of 3D Printing in Drug Delivery. Curr. Pharm. Des..

[49] Chai X., Chai H., Wang X., Yang J., Li J., Zhao Y., Cai W., Tao T., Xiang X. (2017). Fused Deposition Modeling (FDM) 3D Printed Tablets for Intragastric Floating Delivery of Domperidone. Sci. Rep..

[50] Ponsar H., Wiedey R., Quodbach J. (2020). Hot-Melt Extrusion Process Fluctuations and Their Impact on Critical Quality Attributes of Filaments and 3D-Printed Dosage Forms. Pharmaceutics.

[51] Venâncio N., Pereira G.G., Pinto J.F., Fernandes A.I. Influence of the Infill Geometry of 3D-Printed Tablets on Drug Dissolution. Proceedings of the CiiEM 2021.

[52] Gallas M., Boulet P., de Margerie V. (2023). Extrusion for pharma applications: An update. SPE Polym..

[53] Gadgeppa B.O., Tukaram M.S., Baburo G., Sidram G.P., Gaurav A., Agarwal S., Jyoti G., Singal R. (2021). Formulation and Evaluation of 3D Printed Pregabalin Tablets Targeted for Neuropathic Pain by Qbd Approach for Personalized Medicine. Int. J. Pharma Bio Sci..

[54] Kollamaram G., Croker D.M., Walker G.M., Goyanes A., Basit A.W., Gaisford S. (2018). Low Temperature Fused Deposition Modeling (FDM) 3D Printing of Thermolabile Drugs. Int. J. Pharm..

[55] Aguilar-De-Leyva Á., Linares V., Casas M., Caraballo I. (2020). 3D Printed Drug Delivery Systems Based on Natural Products. Pharmaceutics.

[56] Eleftheriadis G.K., Katsiotis C.S., Andreadis D.A., Tzetzis D., Ritzoulis C., Bouropoulos N., Kanellopoulou D., Andriotis E.G., Tsibouklis J., Fatouros D.G. (2020). Inkjet Printing of a Thermolabile Model Drug onto FDM-Printed Substrates: Formulation and Evaluation. Drug Dev. Ind. Pharm..

[57] Alhijjaj M., Belton P., Qi S. (2016). An investigation into the use of polymer blends to improve the printability of and regulate drug release from pharmaceutical solid dispersions prepared via fused deposition modeling (FDM) 3D printing. Eur. J. Pharm. Biopharm..

[58] Mohapatra S., Kar R.K., Biswal P.K., Bindhani S. (2021). Approaches of 3D Printing in Current Drug Delivery. Sens. Int..

[59] Paul A., Sreedevi K., Sharma S.S., Anjana V.N., Thomas S., Ajitha A.R., Jose Chirayil C., Thomas B. (2022). Polylactic Acid (PLA) BT—Handbook of Biopolymers.

[60] Durand B., Marchand C. (2016). Smart Features in Fibrous Implantable Medical Devices. Smart Textiles and Their Applications.

[61] Melocchi A., Parietti F., Maroni A., Foppoli A., Gazzaniga A., Zema L. (2016). Hot-Melt Extruded Filaments Based on Pharmaceutical Grade Polymers for 3D Printing by Fused Deposition Modeling. Int. J. Pharm..

[62] Rastpeiman S., Panahi Z., Akrami M., Haririan I., Asadi M. (2024). Facile Fabrication of an Extended-Release Tablet of Ticagrelor Using Three Dimensional Printing Technology. J. Biomed. Mater. Res. Part A.

[63] Abdollahi A., Ansari Z., Akrami M., Haririan I., Dashti-Khavidaki S., Irani M., Kamankesh M., Ghobadi E. (2023). Additive Manufacturing of an Extended-Release Tablet of Tacrolimus. Materials.

[64] Asadi M., Salehi Z., Akrami M., Hosseinpour M., Jockenhövel S., Ghazanfari S. (2023). 3D Printed PH-Responsive Tablets Containing N-Acetylglucosamine-Loaded Methylcellulose Hydrogel for Colon Drug Delivery Applications. Int. J. Pharm..

[65] Walden D.M., Bundey Y., Jagarapu A., Antontsev V., Chakravarty K., Varshney J. (2021). Molecular Simulation and Statistical Learning Methods toward Predicting Drug–Polymer Amorphous Solid Dispersion Miscibility, Stability, and Formulation Design. Molecules.

[66] Chiou W.L., Riegelman S. (1971). Pharmaceutical Applications of Solid Dispersion Systems. J. Pharm. Sci..

[67] Parulski C., Gresse E., Jennotte O., Felten A., Ziemons E., Lechanteur A., Evrard B. (2022). Fused Deposition Modeling 3D Printing of Solid Oral Dosage Forms Containing Amorphous Solid Dispersions: How to Elucidate Drug Dissolution Mechanisms through Surface Spectral Analysis Techniques?. Int. J. Pharm..

[68] Kissi E.O., Nilsson R., Nogueira L.P., Larsson A., Tho I. (2021). Influence of Drug Load on the Printability and Solid-State Properties of 3D-Printed Naproxen-Based Amorphous Solid Dispersion. Molecules.

[69] Tan D.K., Maniruzzaman M., Nokhodchi A. (2019). Development and Optimisation of Novel Polymeric Compositions for Sustained Release Theophylline Caplets (PrintCap) via FDM 3D Printing. Polymers.

[70] Lasprilla A.J.R., Martinez G.A.R., Lunelli B.H., Jardini A.L., Filho R.M. (2012). Poly-Lactic Acid Synthesis for Application in Biomedical Devices—A review. Biotechnol. Adv..

[71] Da Silva D., Kaduri M., Poley M., Adir O., Krinsky N., Shainsky-Roitman J., Schroeder A. (2018). Biocompatibility, Biodegradation and Excretion of Polylactic Acid (PLA) in Medical Implants and Theranostic Systems. Chem. Eng. J..

[72] Tagami T., Nagata N., Hayashi N., Ogawa E., Fukushige K., Sakai N., Ozeki T. (2018). Defined Drug Release from 3D-Printed Composite Tablets Consisting of Drug-Loaded Polyvinylalcohol and a Water-Soluble or Water-Insoluble Polymer Filler. Int. J. Pharm..

[73] Jamróz W., Kurek M., Czech A., Szafraniec J., Gawlak K., Jachowicz R. (2018). 3D Printing of Tablets Containing Amorphous Aripiprazole by Filaments Co-Extrusion. Eur. J. Pharm. Biopharm..

[74] Fuenmayor E., Forde M., Healy A.V., Devine D.M., Lyons J.G., McConville C., Major I. (2018). Material Considerations for Fused-Filament Fabrication of Solid Dosage Forms. Pharmaceutics.

[75] Kukkonen J., Ervasti T., Laitinen R. (2022). Production and Characterization of Glibenclamide Incorporated PLA Filaments for 3D Printing by Fused Deposition Modeling. J. Drug Deliv. Sci. Technol..

[76] Goyanes A., Allahham N., Trenfield S.J., Stoyanov E., Gaisford S., Basit A.W. (2019). Direct Powder Extrusion 3D Printing: Fabrication of Drug Products Using a Novel Single-Step Process. Int. J. Pharm..

[77] Gottschalk N., Bogdahn M., Quodbach J. (2023). 3D Printing of Amorphous Solid Dispersions: A Comparison of Fused Deposition Modeling and Drop-on-Powder Printing. Int. J. Pharm. X.

[78] Lee V.H., Yamamoto A. (1989). Penetration and Enzymatic Barriers to Peptide and Protein Absorption. Adv. Drug Deliv. Rev..

[79] Krishna R., Garg A., Jin B., Keshavarz S.S., Bieberdorf F.A., Chodakewitz J., Wagner J.A. (2009). Assessment of a Pharmacokinetic and Pharmacodynamic Interaction between Simvastatin and Anacetrapib, a Potent Cholesteryl Ester Transfer Protein (CETP) Inhibitor, in Healthy Subjects. Br. J. Clin. Pharmacol..

[80] Nikam V.K., Kotade K.B., Gaware V.M., Dolas R.T., Dhamak K., Somwanshi S., Khadse A., Kashid V. (2011). Eudragit a Versatile Polymer: A Review. Pharmacologyonline.

[81] Tambuwala M.M., Charbe N.B., McCarron P., Lane M. (2017). Application of Three-Dimensional Printing for Colon Targeted Drug Delivery Systems. Int. J. Pharm. Investig..

[82] Ali H., Kurokawa S., Shehab E., Mukhtarkhanov M. (2023). Development of a Large-Scale Multi-Extrusion FDM Printer, and Its Challenges. Int. J. Lightweight Mater. Manuf..

[83] Kumar R., Kumar M., Chohan J.S. (2021). The Role of Additive Manufacturing for Biomedical Applications: A Critical Review. J. Manuf. Process..

[84] Ventola C.L. (2014). Medical Applications for 3D Printing: Current and Projected Uses. Pharm. Ther..

[85] Akmal J.S., Salmi M., Mäkitie A., Björkstrand R., Partanen J. (2018). Implementation of Industrial Additive Manufacturing: Intelligent Implants and Drug Delivery Systems. J. Funct. Biomater..

[86] Huang W., Zheng Q., Sun W., Xu H., Yang X. (2007). Levofloxacin Implants with Predefined Microstructure Fabricated by Three-Dimensional Printing Technique. Int. J. Pharm..

[87] Halliday A.J., Moulton S.E., Wallace G.G., Cook M.J. (2012). Novel Methods of Antiepileptic Drug Delivery—Polymer-Based Implants. Adv. Drug Deliv. Rev..

[88] Tack P., Victor J., Gemmel P., Annemans L. (2016). 3D-Printing Techniques in a Medical Setting: A Systematic Literature Review. Biomed. Eng. Online.

[89] Wake N., Alexander A.E., Christensen A.M., Liacouras P.C., Schickel M., Pietila T., Matsumoto J. (2019). Creating Patient-Specific Anatomical Models for 3D Printing and AR/VR: A Supplement for the 2018 Radiological Society of North America (RSNA) Hands-on Course. 3D Print. Med..

[90] Pati F., Song T.-H., Rijal G., Jang J., Kim S.W., Cho D.-W. (2015). Ornamenting 3D Ornamenting 3D Printed Scaffolds with Cell-Laid Extracellular Matrix for Bone Tissue Regeneration. Biomaterials.

[91] Tam M.D., Laycock S.D., Bell D.G., Chojnowski A. (2012). 3-D Printout of a DICOM File to Aid Surgical Planning in a 6 Year Old Patient with a Large Scapular Osteochondroma Complicating Congenital Diaphyseal Aclasia. J. Radiol. Case Rep..

[92] Goyanes A., Det-Amornrat U., Wang J., Basit A.W., Gaisford S. (2016). 3D Scanning and 3D Printing as Innovative Technologies for Fabricating Personalized Topical Drug Delivery Systems. J. Control. Release.

[93] Kwak M.K., Jeong H., Suh K.Y. (2011). Rational Design and Enhanced Biocompatibility of a Dry Adhesive Medical Skin Patch. Adv. Mater..

[94] Sodian R., Weber S., Markert M., Rassoulian D., Kaczmarek I., Lueth T.C., Reichart B., Daebritz S. (2007). Stereolithographic Models for Surgical Planning in Congenital Heart Surgery. Ann. Thorac. Surg..

[95] Kim B.-S., Mooney D.J. (1998). Development of Biocompatible Synthetic Extracellular Matrices for Tissue Engineering. Trends Biotechnol..

[96] Bártolo P.J., Chua C.K., Almeida H.A., Chou S.M., Lim A.S.C. (2009). Biomanufacturing for Tissue Engineering: Present and Future Trends. Virtual Phys. Prototyp..

[97] Zadpoor A.A. (2017). Design for Additive Bio-Manufacturing: From Patient-Specific Medical Devices to Rationally Designed Meta-Biomaterials. Int. J. Mol. Sci..

[98] Van Noort R. (2012). The Future of Dental Devices Is Digital. Dent. Mater..

[99] Gargiulo P., Árnadóttir Í., Gíslason M., Edmunds K., Ólafsson I. (2017). New Directions in 3D Medical Modeling: 3D-Printing Anatomy and Functions in Neurosurgical Planning. J. Health Eng..

[100] Liu K., Zhang Q., Li X., Zhao C., Quan X., Zhao R., Chen Z., Li Y. (2017). Preliminary Application of a Multi-Level 3D Printing Drill Guide Template for Pedicle Screw Placement in Severe and Rigid Scoliosis. Eur. Spine J..

[101] Oberoi G., Nitsch S., Edelmayer M., Janjić K., Müller A.S., Agis H. (2018). 3D Printing—Encompassing the Facets of Dentistry. Front. Bioeng. Biotechnol..

[102] Hao W., Liu Y., Zhou H., Chen H., Fang D. (2018). Preparation and Characterization of 3D Printed Continuous Carbon Fiber Reinforced Thermosetting Composites. Polym. Test..

[103] Wu D., Thames J.L., Rosen D.W., Schaefer D. (2013). Enhancing the Product Realization Process with Cloud-Based Design and Manufacturing Systems. J. Comput. Inf. Sci. Eng..

[104] Wu D., Rosen D.W., Wang L., Schaefer D. (2015). Cloud-Based Design and Manufacturing: A New Paradigm in Digital Manufacturing and Design Innovation. Comput. Des..

[105] Akilbekova D., Mektepbayeva D., Kalaskar D.M.B.T. (2017). 5—Patient Specific in Situ 3D Printing. 3D Printing in Medicine.

[106] Zhang Y., Yang Z., Li X., Chen Y., Zhang S., Du M., Li J. (2008). Custom Prosthetic Reconstruction for Proximal Tibial Osteosarcoma with Proximal Tibiofibular Joint Involved. Surg. Oncol..

[107] Tanaka K.S., Lightdale-Miric N. (2016). Advances in 3D-Printed Pediatric Prostheses for Upper Extremity Differences. JBJS.

[108] Salmi M. (2021). Additive Manufacturing Processes in Medical Applications. Materials.

[109] Rosicky J., Grygar A., Chapcak P., Bouma T., Rosicky J. Application of 3D Scanning in Prosthetic and Orthotic Clinical Practice. Proceedings of the 7th International Conference on 3D Body Scanning Technologies.

[110] Bhushan B., Caspers M. (2017). An Overview of Additive Manufacturing (3D Printing) for Microfabrication. Microsyst. Technol..

[111] Weller C., Kleer R., Piller F.T. (2015). Economic Implications of 3D Printing: Market Structure Models in Light of Additive Manufacturing Revisited. Int. J. Prod. Econ..

[112] Kleer R., Piller F.T. (2013). Modeling Benefits of Local Production by Users. Academy of Management Proceedings.

[113] Brabazon P.G., MacCarthy B., Woodcock A., Hawkins R.W. (2010). Mass Customization in the Automotive Industry: Comparing Interdealer Trading and Reconfiguration Flexibilities in Order Fulfillment. Prod. Oper. Manag..

[114] Boschetto A., Bottini L. (2014). Accuracy Prediction in Fused Deposition Modeling. Int. J. Adv. Manuf. Technol..

[115] Goyanes A., Buanz A.B., Hatton G.B., Gaisford S., Basit A.W. (2015). 3D Printing of Modified-Release Aminosalicylate (4-ASA and 5-ASA) Tablets. Eur. J. Pharm. Biopharm..

[116] Griffey J. (2014). The Types of 3-D Printing. Libr. Technol. Rep..

[117] Serajuddin A. (2023). Challenges, Current Status and Emerging Strategies in the Development of Rapidly Dissolving FDM 3D-Printed Tablets: An Overview and Commentary. ADMET.

[118] Chung S., Srinivasan P., Zhang P., Bandari S., Repka M.A. (2022). Development of Ibuprofen Tablet with Polyethylene Oxide Using Fused Deposition Modeling 3D-Printing Coupled with Hot-Melt Extrusion. J. Drug Deliv. Sci. Technol..

